# The Relations Among Prosocial Behavior, Life Satisfaction, and Hedonic Balance Among Young Adults

**DOI:** 10.1111/jopy.70010

**Published:** 2025-08-08

**Authors:** Fulvio Gregori, Belén Lopéz‐Pérez, Tyler Colasante, Giuseppe Corbelli, Tina Malti, Manuel Marti‐Vilar, Cristina Di Giusto Valle, Tamara Benito‐Ambrona, Stefania Sette, Federico Mozzetti, Lucia Manfredi, Maria Gerbino, Concetta Pastorelli, Antonio Zuffianò

**Affiliations:** ^1^ Sapienza, University of Rome Rome Italy; ^2^ University of Manchester Manchester UK; ^3^ Leipzig University Leipzig Germany; ^4^ Uninettuno, Telematic International University of Rome Rome Italy; ^5^ University of Valencia Valencia Spain; ^6^ University of Burgos Burgos Spain; ^7^ Autonoma, University of Madrid Madrid Spain

**Keywords:** hedonic balance, intensive longitudinal data, life satisfaction, prosocial behavior, subjective well‐being

## Abstract

**Objective:**

This study examined the intra‐individual associations between prosocial behavior and two dimensions of subjective well‐being—life satisfaction and hedonic balance—in the daily lives of young adults.

**Method:**

Two samples of Italian and Spanish participants aged 18–35 completed self‐report measures at different intervals: a daily sample assessed for ten consecutive days (*N* = 388; 76% women) and a weekly sample assessed for five consecutive weeks (*N* = 260; 80.3% women). The weekly interval was included as a “sensitivity analysis” to evaluate the stability of effects over longer periods. We employed Random Intercept Cross‐Lagged Panel Models (RI‐CLPMs) to investigate within‐person dynamics while accounting for stable between‐person differences.

**Results:**

In the daily sample, prosocial behavior was associated with increases in life satisfaction from one day to the next one. This effect was not observed across weekly assessments. Although we did not find any empirical evidence that prosocial behavior affects hedonic balance, within‐person correlations between variables were observed in the daily sample, but these tended to disappear in the weekly period.

**Conclusions:**

Incorporating prosocial behaviors into daily routines may promote young adults' life satisfaction. This study contributes to the growing knowledge on how prosocial behavior influences subjective well‐being in everyday life.

Prosocial Behavior (PB)—defined as a voluntary activity intended to benefit another (Eisenberg and Miller 1987)—includes behaviors such as helping, comforting, and sharing things with others (Dunfield 2014; Malti et al. 2016). PB is a common feature of daily life throughout the lifespan (Malti and Speidel 2024; Overbay and Collins 2015). Its positive impacts have been examined at various stages of development, from early childhood to late adulthood (Eisenberg et al. 2015; Zuffianò et al. 2023). For example, PB has been linked to enhanced academic achievement in adolescents (e.g., Caprara et al. 2000) and improved psychological well‐being (e.g., Nantel‐Vivier et al. 2014). Young adults who exhibit prosocial tendencies tend to report elevated levels of self‐esteem (e.g., Zuffianò et al. 2014), while during late adulthood, behaving prosocially (e.g., volunteering) is associated with better physical and mental health (e.g., Yu and Hao 2023).

Of particular importance, a meta‐analysis (Hui et al. 2020) has shown that PB is also positively associated with Subjective Well‐Being (SWB). SWB is a broad concept consisting of different aspects: Life Satisfaction (LS), high positive affect, and low negative affect (Diener 1999). LS refers to a cognitive and global evaluation of one's life, whereas positive and negative affects represent the affective components, the extent to which one experiences pleasant (or unpleasant) emotions. However, most studies that consider the affective aspect of well‐being tend to focus on either positive affect or negative affect, without examining them simultaneously. Hence, the construct of Hedonic Balance (HB), namely the difference between positive affect and negative affect (e.g., Larsen 2000), has been introduced as an index of the affective component of SWB, contributing to filling this gap in the literature.

The literature has shown how people who behave prosocially also reported higher SWB than their counterparts (Hui et al. 2020) across different stages of development, such as childhood and adolescence (e.g., Su et al. 2021), young adulthood (e.g., Layous et al. 2017) and during old age (e.g., Anderson et al. 2010).

From a theoretical point of view, engaging in PB could help improve both aspects of SWB (i.e., LS and HB). The theory of Agency and Communion (Bakan 1966) suggests that behaviors in which individuals act voluntarily, such as PB, might enhance LS and HB by fulfilling the fundamental needs for agency (i.e., the effort to express one's abilities and actively influence one's environment) and communion (i.e., the desire to cultivate social bonds and a sense of belonging). In the same way, the Warm Glow Hypothesis (Andreoni 1990) proposes that the PB itself generates an emotional gratification (called “moral warmth”) that contributes to increasing SWB and motivates people to continue to perform PBs, thus hypothesizing reciprocal relations. Other researchers have focused more specifically on the beneficial role of PB, conceptualizing it as a form of “mood regulation” (Schaller and Cialdini 1988). For instance, empathizing with someone in distress may initially lead to a negative emotional state, which can be alleviated by offering help, thereby restoring HB. Conversely, according to the Mood‐Maintenance Theory (Isen et al. 1987), people with a higher level of HB are more inclined to help others, since this type of behavior allows them to maintain or amplify their equilibrium or positive mood. Similarly, the Broaden‐and‐Build Theory (Fredrickson 2001) suggests that higher levels of positive affect or HB expand cognitive, social, and emotional resources, promoting skills such as empathy and PBs. Overall, these theoretical frameworks support the hypothesis of reciprocal relations between PB and SWB, suggesting that PB might enhance SWB through circular mechanisms involving the fulfillment of agency and communion needs, emotional gratification, and emotional regulation, which in turn promote further PBs aimed at maintaining balanced levels of SWB.

Empirical studies support this idea, indicating PB can foster feelings of belongingness and being valued by others (Zuffianò et al. 2016), which, in turn, helps sustain individuals' happiness (Su et al. 2021). These findings further support the importance of interventions aimed at promoting PB as a beneficial factor across different cultures (Carlo et al. 1999; Eisenberg et al. 2006).

A growing body of research has shown positive, significant, and reciprocal relations between PB and SWB (Hui 2022). Among longitudinal studies, Su et al. (2021), with a large sample of Chinese children assessed three times six months apart, found that PB (e.g., helping classmates) predicted SWB positively 6 months later (*β* ≈0.10), and vice versa (*β* = 0.09), although the influence of PB on SWB was significantly stronger over time. Similarly, in a longitudinal yearly study (Chen et al. 2020), with a sample of Chinese children assessed three times, PB predicted SWB (*β* = 0.19) and vice versa (*β* = 0.10). This suggests that children who helped others during school activities reported greater happiness and satisfaction with school life, while greater well‐being in school also predicted greater prosocial engagement, albeit to a lesser extent.

However, most studies in this field do not differentiate between the two aspects of SWB. This limitation could hinder understanding whether PB is more closely related to LS or HB or whether such associations are similar, making it challenging to design targeted interventions aimed at enhancing perceived well‐being.

## 1 Prosocial Behavior and Life Satisfaction

LS reflects a subjective and global evaluation of one's life and tends to remain stable over time, although it is sensitive to behavioral changes in personal life (Steger and Kashdan 2007). Indeed, longitudinal studies indicate that approximately half of the variance in LS is attributable to personality traits and enduring life circumstances, while the other half reflects situational influences and contextual changes (e.g., Joshanloo 2022).

Several studies have highlighted a positive relation between PB and LS, suggesting that individuals who engage in PBs tend to report a greater LS than their counterparts (e.g., Kushlev et al. 2021; Tandoc Jr et al. 2023).

Regarding yearly longitudinal studies, Jiang et al. (2019) found a positive predictive role of PB (e.g., volunteering) on LS one year later (*b* = 0.08) among older adults, although this effect was small. Other researchers found reciprocal effects between the two variables. For instance, Zhou et al. (2020), with a large sample of adolescents (*M* = 13 years) assessed two times one year apart, found that PB positively predicted LS (*β* = 0.44) and vice versa (*β* = 0.10), although the effect of LS on PB was significantly smaller. However, it is important to highlight that most long‐term longitudinal studies have not separated within‐person and between‐person variance, making it difficult to determine whether observed effects reflect changes over time within individuals (within‐person) or stable differences between individuals (between‐person).

In this regard, some recent studies using intensive longitudinal data have attempted to disentangle these two sources of variance to gain a more precise understanding of the underlying processes. For instance, Zuffianò et al. (2018), in a daily study involving young adults, found a positive predictive role of PB (i.e., helping) on LS at the within‐person level (*β* = 0.33). Similar results were found also using Ecological Momentary Assessment (EMA) among adolescents (*β* = 0.13) and adults (*β* = 0.29; Gregori et al. 2025). These results indicated that if a person behaved more prosocially than usual at a specific moment, a higher than usual level of LS was reported subsequently.

## 2 Prosocial Behavior and Positive Emotions

Positive affect refers to the extent to which an individual subjectively experiences a positive mood (Peterson 2006). It reflects pleasant and positive experiences. Despite its significant overlap with the concept of positive emotions (e.g., happiness), the two constructs are not identical. Positive affect is more closely related to mood states, whereas positive emotions involve both momentary positive feelings and characteristic patterns of physiological arousal, thoughts, and behaviors (Peterson 2006). However, in this article, the two elements are treated interchangeably as they denote affective indices of SWB.

Numerous studies have highlighted a strong relation between PB and positive emotions, showing that individuals who engage in helping, sharing, or supporting behaviors tend to report higher levels of emotional well‐being across various contexts and age groups (Carlson et al. 1988; Martela and Ryan 2016; Varma et al. 2023). Additionally, some research suggests the existence of reciprocal effects between the two variables, indicating that not only does PB foster positive emotions, but also experiencing positive emotions, such as happiness, can encourage PB (Aknin et al. 2012; Kushlev et al. 2021).

The relations between PB and positive affect were also investigated through a longitudinal perspective. In a monthly study involving assessments of workers, it was observed that PB (i.e., prosocial spending) significantly predicted increased happiness six weeks later (*β* = 0.81; Dunn et al. 2008). In the same way, (Thoits and Hewitt 2001) assessed elder participants twice (3 years between the first and the second measurement) and found that PB (i.e., hours of volunteering work) predicted positive affect (*β* = 0.19) and vice versa (*β* = 0.01), although the latter effect was very small. Similarly, the recent yearly longitudinal study of Yu and Hao (2023), with a sample of children, showed a positive predictive role of PB on both self‐reported happiness (*β* = 0.045) and peer‐reported happiness (*β* = 0.26). Regarding peer‐reported happiness, a reciprocal effect was also found (although this effect was somewhat weaker).

Other authors have studied the relations between the two variables using an intensive longitudinal perspective, distinguishing between within‐person and between‐person components of variability. The study of Rinner et al. (2022) using EMA (6 times daily across one week) with a sample of adults showed a significant and positive within‐person association from PB to happiness (*b* = 0.08), highlighting how PB has a positive role in people's daily lives. Another intensive longitudinal study (Snippe et al. 2018) which considered a sample of adults assessed with EMA (three times a day across thirty days) found small yet significant within‐person reciprocal positive effects between PB and positive affect (i.e., from positive affect to PB *β* = 0.06; from PB to positive affect *β* = 0.11).

In summary, research on the relation between PB and positive affect has shown that these two variables are interconnected both in the short and long term, with evidence suggesting they might even influence each other over very short periods (e.g., within hours; Hui 2022). However, it is important to acknowledge that, although the literature reviewed above has primarily focused on the beneficial associations between PB and positive affect, a substantial body of literature has also investigated the protective role of PB on negative affect (e.g., Chi et al. 2021; Hui and Kogan 2018), an aspect that was not explored in the present work.

## 3 The Present Study

Drawing on the previously mentioned literature (e.g., Gregori et al. 2025; Hui 2022; Zuffianò et al. 2018), we believe that our study advances the body of knowledge in this field in at least three different ways. Firstly, we examine the relations between PB and LS/HB across two different time lags: daily and weekly. To our knowledge, there is a gap in the literature regarding studies that consider simultaneously and compare samples of young adults across different time lags such as these. From a theoretical point of view, we expect that these relations might be stronger in the short term (i.e., at the daily level), as the fulfillment of needs (e.g., agency and communion needs; Bakan 1966) is often tied to immediate social experiences and situational feedback. Daily measurements are likely to capture the more direct and proximal effects of engaging in prosocial behavior on well‐being, while weekly assessments may reflect more diluted or aggregated patterns. Testing on a slightly longer interval (i.e., weekly) serves as a longitudinal robustness check, assessing whether PB in one week extends to LS or HB the next.

Secondly, rather than focusing solely on positive or negative affect as components of SWB, we include HB as an index of hedonic well‐being that takes into account both positive and negative affect simultaneously. Although some intensive longitudinal studies of young adults have already investigated the relations between PB and positive affect (Rinner et al. 2022; Snippe et al. 2018), to the best of our knowledge, no research has yet examined the relations between PB and HB. Considering HB as the affective component of SWB is important, as positive affect alone does not necessarily indicate an absence of negative affect and vice versa (Larsen 2000). Specifically, we expected that higher levels of PB would be associated with higher levels of HB on the following day.

Finally, we distinguished between within‐person and between‐person levels to better understand the relations from PB on LS/HB in daily life (within‐person level) while controlling for individual differences (between‐person). Specifically, we hypothesized that more‐than‐usual levels of PB would be associated with more‐than‐usual levels of LS/HB on the following day. To answer this research question, we clarified at which level the relations among PB, LS, and HB occurred (i.e., between‐person level reflecting trait‐like or stable/enduring interpersonal differences in PB, LS, and HB; and the within‐person level reflecting state‐like or occasion‐specific fluctuations in these variables).

To address our research questions, we conducted four Random Intercept Cross‐Lagged Panel Models (RI‐CLPMs; Hamaker et al. 2015) across a daily and weekly sample of young adults. In the daily sample, participants reported their PB, LS, and HB (i.e., recording their positive and negative affect) once per day over 10 consecutive days. Within the daily sample, we estimated two RI‐CLPMs: one model includes daily PB and LS, and another includes daily PB and HB. In the weekly sample, data were collected once a week over 5 consecutive weeks, and we estimated two RI‐CLPMs: one model with PB and LS, and the other with PB and HB.

## 1 Method

### 1.1 Samples and Design

Following the indications offered by Maas and Hox (2005) for nested data, we recruited at least 50 level‐2 units (i.e., participants) in both our daily and weekly samples.

### 1.2 Daily Sample

Participants were 338 young adults (university students ages ranged from 18 to 35 years) enrolled in a university degree in Italy (*n* = 101, 91% women, *m*_age_ = 22.35, SD = 2.43) and Spain (*n* = 237, 76% women, *m*_age_ = 21.72, SD = 2.52). Data were collected during the academic years 2017–2018 and 2018–2019, and participants were compensated with partial course credit. Before engaging in the daily questionnaire, participants provided informed consent. The data collection involved participants responding to the questionnaire once a day for ten consecutive days, utilizing an online platform (Qualtrics). Participants could complete the daily questionnaire in the evening between 8:00 p.m. and 11:59 p.m. The items were adapted for a daily measure beginning with the phrase “Thinking about today…”. On average, participants responded 7 days out of 10. With 73.4% of the participants providing data for at least 60% (6/10 days), the retention rate was considered quite high.

### 1.3 Weekly Sample

A total of 260 young adults, aged from 18 to 35 years, took part in the study. They were students pursuing a university degree in Italy (*n* = 108, 88.9% women, *m*_age_ = 22.18, SD = 2.66) and Spain (*n* = 152, 80.3% women, *m*_age_ = 22.53, SD = 3.73). The data collection occurred during the academic years 2017–2018 and 2018–2019, and also in this case, participants received partial course credit as compensation. Before engaging in the daily questionnaire, participants provided informed consent. The questionnaires were administered through an online platform (Qualtrics), once a week for a total of 5 weeks. The items were adapted for a weekly measure beginning with “Last week…”. On average, participants completed weekly surveys for 3 out of 5 weeks. The retention rate of the weekly sample was considered again quite high: 76% provided data for at least 3/5 weeks (60%).

## 2 Measures

### 2.1 Prosocial Behavior

PB was measured by the 16‐item Prosocial Behavior Scale (PBS; Caprara et al. 2005), which captures the dimension of prosociality, reflecting in the three prototypical prosocial behaviors: helping (e.g., “I am pleased to help my friends/colleagues in their activities.”), comforting (e.g., “I try to console those who are sad”) and sharing (e.g., “I share the things that I have with my friends”). Participants responded on a 5‐point Likert scale (From 1 = *never/almost never* to 5 = *always/almost always*). The scale showed reliability (Cronbach's alpha) ranging from *α* = 0.88 to *α* = 0.95 in the daily sample and from *α* = 0.90 to *α* = 0.97 in the weekly sample.

### 2.2 Life Satisfaction

LS was assessed using the 5‐item set of the “Satisfaction with Life Scale” (Diener et al. 1985) with a 7‐point Likert scale (from 1 = *strongly disagree* to 7 = *strongly agree*). The scale was designed to evaluate the cognitive component of SWB (e.g., “I am satisfied with my life”). This scale showed a Cronbach's alpha ranging from *α* = 0.86 to *α* = 0.95 in the daily sample and from *α* = 0.89 to *α* = 0.95 in the weekly sample.

### 2.3 Hedonic Balance

HB was measured using the “Positive and Negative Affect Scale” (PANAS; Watson et al. 1988). PANAS assesses two distinct dimensions, Positive Affect (PA) and Negative Affect (NA), with a 5‐point Likert scale (1 = *very slightly or not at all*, 5 = *extremely at all*). Both scales have 10 items (e.g., PA= “Today I felt happy”, NA “Today I felt sad”). HB was obtained by the difference between PA and NA scores and therefore ranged between −4 (absence of PA and presence of NA) and + 4 (absence of NA and presence of PA; i.e., a better hedonic balance). Cronbach's alpha was calculated separately for each dimension. Specifically, for PA, reliability had a minimum value of *α* = 0.75 and a maximum value of *α* = 0.85 in the daily sample and a minimum value of *α* = 0.77 and a maximum value of *α* = 0.84 in the weekly sample. Similarly, for NA, reliability had a minimum value of *α* = 0.83 and a maximum value of *α* = 0.90 in the daily sample and a minimum value of *α* = 0.83 and a maximum value of *α* = 0.88 in the weekly sample.

### 2.4 Data Analytic Approach

To better understand the relation between PB and LS on one side, and PB and HB on the other side, we employed a RI‐CLPM (Hamaker et al. 2015). A RI‐CLPM breaks down the score of participant *i* at time t regarding the variable of interest (e.g., PB) into three distinct components. To do so, first, it considers the group mean (*μ*_*t*_) of PB at a specific time point. Then, it takes into account the stable, time‐invariant trait‐like deviation from the group mean, which is represented by the latent random intercept (*κ*_*i*_). Lastly, it incorporates the momentary deviation (*ρ*_*it*_) of each participant at a given time point. In essence, the measured score of PB is expressed as the sum of three components:

$${\mathrm{PB}}_{\mathrm{it}}=\mu_{t}+\kappa_{i}+\rho_{\mathrm{it}}$$

**FIGURE 1:**
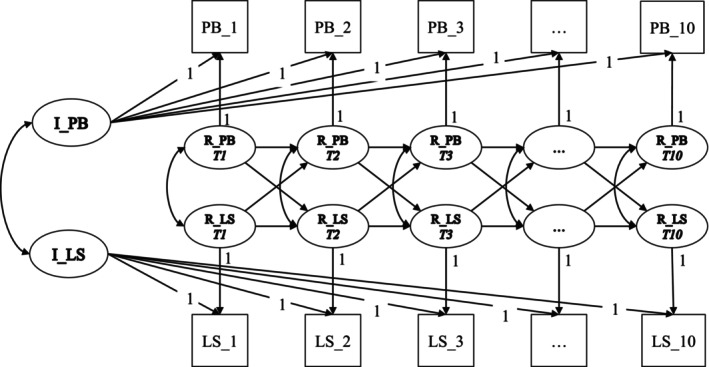
Example of random intercept cross‐lagged panel model (RI‐CLPM). In the figure, prosocial behavior (PB) and life satisfaction (LS). To simplify, the diagram exclusively illustrates the Random Intercept Cross‐Lagged Panel Model (RI‐CLPM) in which the variables Prosocial Behavior (PB) and Life Satisfaction (LS) within the daily sample. At the between‐person level (trait‐like), the figure shows the estimated correlation (↔) between the latent random intercepts (I) of I_PB and I_LS. At the within‐person level (state‐like) the figure reports: Correlations (↔) between the residual components of PB (R_PB) and LS (R_LS) from time 1 (T1) to time 10 (T10); carry‐over effects (→) for R_PB and R_LS; spillover effects (→) from R_PB(t‐1) to R_LS(t), and from R_LS(t‐1) to R_PB(t).

RI‐CLPM, therefore, permits the separation of between‐person associations (i.e., trait‐level: did participants who, on average, reported higher PB across the ten days/five weeks also reported higher LS/HB than their counterparts?) from within‐person associations (i.e., state‐level: did participants who, in a specific moment, had higher PB also reported higher LS/HB than their counterparts at the same/next occasion?). In particular, when examining deviations from expected levels among participants, three distinct relations can be explored at the individual level, as outlined by Hamaker et al. (2015): (a) simultaneous, time‐specific covariations (e.g., were higher/lower than expected levels of PB related to higher/lower than expected levels of LS/HB at the same day/week?); (b) carry‐over effects (the autoregressive parameters; i.e., did participants with higher/lower than expected levels of PB on a given day/week report higher/lower than expected levels of PB at the next day/week?); and (c) spillover effects (the cross‐lagged parameters; i.e., did higher/lower than expected levels of PB on a given day/week predict higher/lower than expected levels of LS/HB at the next day/week and vice‐versa?). In addition, we examined the consistency of each within‐person effect over time. This was done by comparing the fit of two models: the unconstrained RI‐CLPM, in which within‐person effects were allowed to vary freely over time, and the constrained RI‐CLPM, where the same parameters were constrained to be equal across time intervals (Mulder and Hamaker 2021). We utilized the chi‐square difference test for nested models (Δ*χ* ^2^) to assess the significance of any differences in fit between the two models. Figure 1 shows the RI‐CLPM model graphically. Finally, as indicated by Mulder and Hamaker (2021) we performed an additional RI‐CLPM to test the robustness of the effects while controlling for both time‐invariant covariates (i.e., age, gender, and country).

We assessed the appropriateness of our models by employing conventional criteria commonly used in structural equation modeling (Kline 2023). Specifically, in addition to a non‐significant *χ* ^2^, we considered Comparative Fit Index (CFI) and Tucker‐Lewis Index (TLI) values exceeding 0.95, and Root‐Mean‐Square‐Error‐of‐Approximation (RMSEA) values (along with its 90% confidence interval) below 0.08 as indicative of a well‐fitting model. All model parameters were estimated using maximum likelihood with robust standard errors (MLR) in Mplus 8.4 (Muthén and Muthén 1998–2017).

## 3 Results

Syntaxes, outputs, and datasets are uploaded in the OSF at the following link: https://osf.io/s3jv5/?view_only=2ab61425382240f1ae5c12ecfba9824f.

### 3.1 Preliminary Analyses

Tables S1 and S2, reported in the Supporting Information, show the correlations between PB and LS/HWB over time. Specifically, Table S1a and Table S1b refer to the daily sample, while Table S2a,b refer to the weekly sample. The variables are (almost) all positively and moderately correlated in both samples. To examine whether meaningful differences existed between the within‐ and between‐person levels, ICCs were calculated for all variables included in the models. The results indicated a balanced distribution of variance across the two levels (i.e., approximately 50%). In addition, in line with the methodological recommendations of Cooke et al. (2022), Multilevel Confirmatory Factor Analyses (MCFA) were conducted for each component of subjective well‐being to assess their structural validity. All scales (PB, LS, positive affect, and negative affect) showed acceptable fit indices.

### 3.2 Ri‐CLPM

#### 3.2.1 Daily Sample

Regarding the model in which we considered PB and LS (Model 1), the constrained RI‐CLPM in which the unstandardized within‐person parameters were constrained to equality over time showed a good fit, *χ* ^2^(181) = 264.68, *p* < 0.001, CFI = 0.97, TLI = 0.96, RMSEA = 0.04 (90% CI: 0.027, 0.046) and was not statistically different, Δ*χ* ^2^(40) = 34.97, *p* = 0.696, from the unconstrained RI‐CLPM. Hence, the size of the time‐specific covariations, carry‐over effects, and spillover effects remained stable for 10 days. As reported in Table 1, the results of the constrained RI‐CLPM highlighted a positive correlation between the latent random intercept of PB and that of LS (*r* = 0.28; *p* < 0.001), highlighting how the association between the two daily variables is partly due to interindividual differences.

**TABLE 1:** Summary of standardized parameters for daily model: Model 1 (prosocial behavior and life satisfaction), and Model 2 (prosocial behavior and hedonic balance).

Model 1: PB–LS			Model 2: PB–HB		
Parameters	Std	*p*	Parameters	Std	*p*
*Between‐level*			*Between‐level*		
I_PB ↔ I_LS	0.280	< 0.001	I_PB ↔ I_HB	0.118	NS
*Within‐level correlations*			*Within‐level correlations*		
R_PB ↔ R_LS_T1–T10_	0.207 to 0.386	< 0.001 to < 0.05	R_PB ↔ R_HB_T1–T10_	0.068–0.338	< 0.001 to NS
*Carry‐over effects*			*Carry‐over effects*		
R_PB_t_ → R_PB_t+1_	0.278 to 0.352	< 0.001	R_PB _t_ → R_PB_t+1_	0.272–0.337	< 0.001
R_LS_t_ → R_LS_t+1_	0.150 to 0.206	< 0.001	R_HB _t_ → R_HB_t+1_	0.221–0.304	< 0.001
*Spillover effects*			*Spillover effects*		
R_PB _t_ → R_LS_t+1_	0.068 to 0.096	< 0.05	R_PB _t_ → R_HB_t+1_	0.006–0.008	NS
R_LS _t_ → R_PB_t+1_	–0.008 to −0.011	NS	R_HB _t_ → R_PB_t+1_	0.004–0.006	NS

*Note:* The ranges reported refer to effects observed across Time 1 to Time 10. Std. = standardized coefficient; NS = nonsignificant; I_LS=Intercept of Life Satisfaction; I_PB=Intercept of Prosocial Behavior; R_PB_T_ and R_PB_T+1_ = Range of the within component of Prosocial Behavior from day 1 to day10; R_LS_T_ … R_LS_T+1_ = Range of the within component of Life Satisfaction from day 1 to day 1.

Similarly, at the state level (within‐person), we found a similar pattern of positive time‐specific correlations (Except for T1, where the association between these two variables at the within level was non‐significant; see Table 1). Specifically, higher‐than‐expected levels of PB on a given day were related to higher‐than‐expected levels of LS (*rs* ranged from 0.207 to 0.386, *p*s < 0.05). We also found positive and statistically significant day‐to‐day carry‐over effects of PB (*β* 0.30, *p* < 0.001) and LS (*β* ≈0.17, *p* < 0.001). Moreover, the result of Model 1 showed spillover effects of PB on LS, indicating that higher‐than‐expected levels of PB on a given day were associated with slightly higher‐than‐expected levels of LS on a subsequent day (*β* ≈0.07, *p* < 0.05); however, the spillover effects of LS on PB were not significant (*p* > 0.05). This means that on days when PB occurs, LS is elevated and carries over into the following day. However, this elevated LS does not appear to motivate additional PB the next day.

Finally, we tested the robustness of these results while controlling for the covariates of gender, age, and country (Italy vs. Spain). This RI‐CLPM showed a good fit *χ* ^2^(181) = 254.99, *p* < 0.001, CFI = 0.97, TLI = 0.96, RMSEA = 0.04 (90% CI: 0.024, 0.045). The results indicated that the within‐person associations between PB and LS did not differ from those observed in the RI‐CLPM model without covariate control. This finding indicates that, regardless of gender, age, and country, the previously found relations between the two variables remain stable.

Considering the model in which we included PB and HB (Model 2), the constrained RI‐CLPM showed a good fit, *χ* ^2^(181) = 253.63, *p* < 0.001, CFI = 0.971, TLI = 0.97, RMSEA = 0.034 (90% CI: 0.024, 0.044), and was not statistically different, *Δχ* ^2^(40) = 40.13, *p* = 0.46, from the previous unconstrained RI‐CLPM. Hence, the size of the time‐specific covariations, carry‐over effects, and spillover effects remained the same across 10 days. As shown in Table 1, the findings from the constrained RI‐CLPM revealed a slight yet significant positive correlation between the latent random intercept of PB and that of HB (*r* = 0.12; *p* < 0.001), indicating that the relation between the variables showed a small correlation in terms of stable inter‐individual differences.

At the state‐level (within‐person), we found a pattern of positive within‐person correlations that were approximately constant across time, suggesting that the relation between the two variables might also be due to the state‐level and not only due to stable personality characteristics. More in detail, higher‐than‐expected levels of PB on a given day were related to higher‐than‐expected levels of HB (*rs* ranged from 0.249 to 0.338, *p*s < 0.005). We also found positive and statistically significant day‐to‐day carry‐over effects for PB (*β* ≈0.30, *p* < 0.001) and HB (*β* ≈0.26, *p* < 0.001; see Table 1). The results of Model 2 did not show any significant spillover effect.

For more details on the results of the models tested in the daily sample, we invite the reader to consult the Supporting Information, which includes Figure S1a (model representation with PB and LS and related results), Figure S1b (model with PB and HB and results), and Table S3 (which provides a detailed report of all analytical results).

#### 3.2.2 Weekly Sample

As the weekly data were collected over a longer period than one month (5 weeks), in the preliminary phase, we also used the latent curve model (Bollen and Curran 2006) to ensure that there was no trend in the variable over time (changes in the means of PB, LS and HB). To determine the trajectory of each variable (i.e., PB, LS, and HB), we specifically evaluated no‐growth, linear, quadratic, and cubic models. The results showed that neither PB nor LS had a significant trend (only HB showed a linear decline). To further assess the robustness of our results, we also tested the Latent Curve Model with Structured Residuals (LCM‐SR). Recent literature has recommended this approach as a more appropriate alternative to traditional CLPMs when modeling interindividual differences and developmental dynamics (Berry and Willoughby 2017; Mund and Nestler 2019). The findings were virtually identical. The outputs are available at the following OSF link: https://osf.io/s3jv5/?view_only=2ab61425382240f1ae5c12ecfba9824f.

Regarding the model in which we considered PB and LS (model 3), the chi‐square difference between the constrained and unconstrained RI‐CLPM was significant, suggesting that the unconstrained model was the most appropriate and showed the following fit indices *χ* ^2^(21) = 25.35, *p* = 0.23, CFI = 0.99, TLI = 0.99, RMSEA = 0.03 (90% CI: 0.000, 0.062). At the between‐person level, a positive and significant association was observed between PB and LS (*r* = 0.34; *p* < 0.01), indicating that, with a weekly time lag, the relation between the two constructs seems due to dispositions rather than to momentary fluctuations. In fact, at the within‐person level, correlations between the two variables varied from week to week, with some weeks showing significant correlations while others did not, indicating fluctuating patterns over time. Both carry‐over and spillover effects showed a similar inconsistent pattern, suggesting that a positive fluctuation in PB/LS one week did not always predict a positive fluctuation in PB/LS the following week and vice versa (see Table 2).

**TABLE 2:** Summary of standardized parameters for weekly models: Model 3 (prosocial behavior and life satisfaction), and Model 4 (prosocial behavior and hedonic balance).

Model 3: PB–LS			Model 4: PB–HB		
Parameters	Std	*p*	Parameters	Std	*p*
*Between‐level*			*Between‐level*		
I_PB ↔ I_LS	0.335	< 0.01	I_PB ↔ I_HB	0.397	< 0.001
*Within‐level correlations*			*Within‐level correlations*		
R_PB ↔ R_LS_T1–T10_	−0.211 to 0.459	< 0.001 to NS	R_PB ↔ R_HB_T1–T10_	−0.271 to 0.454	< 0.001 to NS
*Carry‐over effects*			*Carry‐over effects*		
R_PB_t_ → R_PB_t+1_	−0.333 to 0.608	< 0.001 to NS	R_PB_t_ → R_PB_t+1_	−0.353 to 0.581	< 0.001 to NS
R_LS_t_ → R_LS_t+1_	0.125 to 0.553	< 0.001 to NS	R_HB_t_ → R_HB_t+1_	−0.102 to 0.288	< 0.05 to NS
*Spillover effects*			*Spillover effects*		
R_PB_t_ → R_LS_t+1_	−0.234 to 0.146	NS	R_PB_t_ → R_HB_t+1_	−0.286 to 0.253	< 0.05 to NS
R_LS_t_ → R_PB_t+1_	−0.283 to 0.165	NS	R_HB_t_ → R_PB_t+1_	−0.275 to 0.115	NS

*Note:* The ranges reported refer to effects observed across Time 1 to Time 10. Std. = standardized coefficient; NS = nonsignificant; I_LS = Intercept of Life Satisfaction; I_PB = Intercept of Prosocial Behavior; R_PB_T_ and R_PB_T+1_ = Range of the within component of Prosocial Behavior from day 1 to day10; R_LS_T_ … R_LS_T+1_ = Range of the within component of Life Satisfaction from day 1 to day 1.

Also concerning model 4, in which we considered PB and HB, the significant chi‐square difference between the constrained and unconstrained RI‐CLPM indicates that the unconstrained model was the most acceptable and showed the following fit indices *χ* ^2^(21) = 30.82, *p* = 0.08, CFI = 0.99, TLI = 0.97, RMSEA = 0.04 (90% CI: 0.000, 0.073). The results showed a pattern similar to those emerging from model 3 (see Table 2), suggesting that the emotional component of SWB was also associated with PB, particularly in terms of stable differences among individuals (*r* = 0.40; *p* < 0.001).

At the within‐person level, the correlations between the two variables changed weekly, with some weeks showing significant associations while others did not, highlighting fluctuating patterns over time. Similarly, both carry‐over and spillover effects followed the same trend, suggesting that within‐person changes in PB/HB one week were not associated with fluctuations in PB/HB in the following week (see Table 2).

Further information on the results of the models tested in the weekly sample can be found in the Supporting Information, which includes Figure S2a (depicting the model with PB and LS and corresponding results), Figure S2b (model with PB and HB and related results), and Table S4 (offering a comprehensive summary of the analytical findings).

#### 3.2.3 Additional Analysis

To strengthen the robustness of our analyses based on the daily sample, we conducted additional RI‐CLPM analyses examining PB in relation to positive and negative affect separately. Specifically, two distinct models were tested: one including only PB and positive affect, and the other including only PB and negative affect. As in the main analyses of the study, both unconstrained and constrained models were estimated, and chi‐square indices were compared. The results were largely consistent with those obtained using the HB index, suggesting that the effects of PB on well‐being indicators are stable across different operationalizations. More specifically, increases in PB on a given day were not associated with subsequent decreases in negative affect or increases in positive affect on the following day.

## 4 Discussion

Numerous studies have consistently evidenced a robust association between PB and SWB (e.g., Hui et al. 2020). Moreover, investigations have extended to examining the relations between PB and the sub‐dimensions of SWB (i.e., LS and positive affect). However, research investigating the relations between PB and the affective component of SWB mostly focused on positive or negative affect. Therefore, we aimed to assess how PB was associated with HB (i.e., the ratio of positive to negative affect; Larsen 2000), an indicator of emotional well‐being that takes into account both positive and negative affect.

In our study, we wanted to examine if intra‐individual fluctuations in PB were associated with positive peaks in LS/HB, controlling for individual differences in behaving prosocially and experiencing LS/HB (between‐person). Moreover, we aimed to explore whether, within the sample spanning shorter (daily) or broader (weekly) time intervals, the relations among the variables examined (both at the between and within levels) stayed consistent or underwent changes. It is important to underline that, although the present study was mainly exploratory, it was based on solid theoretical assumptions that allowed us to expect reciprocal effects between PB and SWB. Theories suggest a circular relation between SWB and PB. Specifically, the perceived well‐being expands cognitive and social resources that encourage PB (Fredrickson 2001; Isen et al. 1987), while the emotional reward of helping others—the “warm glow” (Andreoni 1990)—boosts SWB and motivates further PB.

Interestingly, as extensively described in the introduction of this study, intensive longitudinal studies (e.g., daily) have found that PB has a stronger effect on SWB and its components, LS and HB, while the effects from SWB to PB, as well as from LS/HB to PB at the next time point, appear weaker or null. Although this study had an exploratory nature, the results suggest a stronger spillover effect from PB to LS and HB than in the opposite direction.

Regarding the daily sample, the RI‐CLPM in which PB and LS were considered showed how the relations between the two variables occurred both at the trait and state levels. As expected, at the between‐person level, young adults who reported higher levels of PB also reported higher levels of LS than their counterparts, consistent with previous studies (e.g., Ng and Diener 2022). Similarly, at the within‐person level, the days when young adults behaved more prosocially than usual also reported a positive deviation in LS from their average, highlighting a positive association between PB and LS in young adults' daily lives (see also Zuffianò et al. 2018). Moreover, the model showed a persistence of these variables from one day to the next (carry‐over effects), indicating that a positive fluctuation in PB or LS one day tended to carry over to the following day. Regarding the relation between variables from a time to the subsequent (i.e., spillover effect), the findings indicated that a positive fluctuation in PB on a given day was associated with a positive fluctuation in LS the subsequent day, supporting that the benefit of being more prosocial than usual on a given day might carry over to the next. This may be due to the fact that, in everyday contexts, when a young adult behaved more prosocially than usual (e.g., helped or comforted more on a certain day), they were able to satisfy their needs for agency and communion (Bakan 1966), feeling more immediately satisfied with their own life. Specifically, according to Ryff and Singer's (2008) multidimensional model of psychological well‐being, prosocial behavior can promote individuals' sense of purpose and personal growth by reinforcing their perception of being valuable and contributing to society. Although LS is typically framed within the hedonic view, it can also reflect, at least in part, eudaimonic dimensions such as meaning and fulfillment. Engaging in prosocial acts may thus enhance LS by fostering a deeper sense of coherence and alignment with personal values. No spillover effects emerged in the opposite direction (i.e., a positive fluctuation in LS experienced on a day did not predict positive fluctuation in PB the following day). This nonsignificant effect aligns with previous studies showing that daily fluctuations in PB tend to be associated with increases in LS, rather than the reverse (e.g., Zuffianò et al. 2018). Although the relation between LS and PB may be influenced by factors such as sense of meaning or emotional regulation (Zuffianò et al. 2018), its direct short‐term influence on daily life may be weak or absent (Kahana et al. 2013). This suggests that interactions between PB and LS might occur within even shorter time frames, such as those captured by EMA (e.g., hours; Gregori et al. 2025), which better capture moment‐to‐moment fluctuations in well‐being.

The results of the model were stable while controlling for gender, age, and country (i.e., Spain and Italy), supporting the robustness of the phenomenon.

Regarding the daily RI‐CLPM in which PB and HB were considered, the results showed how the relations between the two variables occur at the trait and state levels. At the between‐person level, young adults who reported higher levels of actions such as helping or comforting, on average, also reported higher levels of HB. Regarding the within‐person level, the days on which participants behaved more prosocially than usual also reported a positive deviation in HB. The carry‐over effects showed the persistence of PB/HB from one day to the next. This suggests that if one day a participant had a positive deviation of PB/HB, this deviation was carried over to the next day. No spillover effects were observed from the RI‐CLPM, indicating that at the within‐person level, a momentary intra‐individual fluctuation in PB was not associated with fluctuations in HB the next day and vice versa (we only found same‐day relations between PB and HB). These results were also confirmed by additional models in which positive and negative affect were considered as separate variables related to PB. A possible explanation for this result could be the dynamic and fluctuating nature of emotions, which might be more ephemeral than LS's judgments that, even at the daily level, reflect a global cognitive appraisal that might be less sensitive to momentary fluctuations. Consequently, a daily framework may not adequately capture the beneficial effect of PB on the affective dimension of well‐being and vice versa, as it could instead occur when considering a time window of a few hours (Gregori et al. 2025; Rinner et al. 2022; Snippe et al. 2018).

As for the weekly sample, in which PB and LS were considered as variables, the main result that emerged is the association between the two intercepts. This means that individuals who reported higher levels of PB over the five weeks also reported higher levels of LS than their counterparts. At the within‐level, however, within‐person correlations were not consistently significant throughout the weeks, indicating that weeks in which young adults reported a positive deviation in PB from their average sometimes also reported a positive deviation in LS, but not always. This result may suggest that different factors might influence this association during the week, such as individual needs (Snippe et al. 2018), perceived social support at specific times (Thoits 2011), and motivations for prosocial behavior (Weinstein and Ryan 2010). In the same way, even carryover effects did not show significant results over time, suggesting that if participants in the study behaved more prosocially than usual or experienced an increase in LS compared to their average in one week, this deviation was not always carried over to the following week. Moreover, spillover effects did not yield significant results over the 5 weeks. This suggests that a tighter time frame (daily or even multiple times per day) may be best to capture “real‐life” relations of these constructs (e.g., Gregori et al. 2025).

Similarly, in the RI‐CLPM in which PB and HB were considered, the main result was the correlation between the two intercepts at the between‐person level: Individuals who reported higher levels of PB also reported higher levels of HB than their counterparts. At the within level, the correlations were not consistently significant over time, indicating that weeks in which individuals reported a positive deviation in PB from the mean level sometimes reported a positive deviation in HB, but this was not observed at each time point. This suggests that probably other factors may influence the relation between PB and HB every week. Similarly, carry‐over effects were not significant, indicating that increases in PB or HB within one week were not always sustained into the following week. Also, spillover effects were nonsignificant over the five weeks, supporting the hypothesis that, at the within‐person level, PB might have an impact on the affective component of SWB when considered within a very short time frame (e.g., a few hours).

## 5 Limitations and Conclusion

Future research should address a number of the limitations identified in our study. First, we recognize that the study might have lacked sufficient power to detect small effects such as those observed in our research, and future studies should aim to recruit larger sample sizes to enhance statistical power.

Second, the absence of pre‐registered hypotheses, as our aims were primarily exploratory. Future studies could benefit from pre‐registered hypotheses and testing models based on empirical evidence.

Third, we acknowledge the conceptual limitations of the HB operationalization. Although our study constitutes a contribution to the ongoing discussion about how to best capture the dynamic interplay between affective states in everyday life, especially in the daily lives of young adults, the practical implications of considering HB as a measure for well‐being require further empirical evidence.

The fourth limitation concerns the use of self‐report measures to evaluate the variables of interest. While self‐report measures are valuable for daily and weekly investigations, results would be strengthened if they included information from other sources, such as friends or colleagues. Thus, future studies should consider using multi‐informant methods to assess PB.

Fifth, future research should examine the role of other factors that might moderate the relation between PB and LS/HB, such as the recipients of help (e.g., partner, friends, or a family member, etc.; Gregori et al. 2025), the type of specific PB (helping, comforting, sharing; Dunfield 2014) and the needs that are satisfied by PB, such as the need for autonomy, relatedness, or competence (Ryff and Singer 2008).

Sixth, our study considered daily and weekly data of LS/HB and PB, which may somehow limit the generalizability and interpretation of the results. Future research could explore a variety of time lags, for instance, using an EMA approach, where measurements are taken within a few hours, or employing yearly longitudinal data.

Moreover, although the RI‐CLPM has shown that within‐level PB was associated with LS, future studies could treat PB as an instrumental variable, using the Within‐Person Encouragement Design (Schmiedek and Neubauer 2020) to address the effect of unmeasured confounders (Antonakis et al. 2010).

Finally, it is important to consider that the relation between PB and LS/HB may be influenced by several time‐varying confounders, such as perceived social support at specific points in time (Thoits 2011) and the motivations underlying PB (Weinstein and Ryan 2010). Future research should incorporate these time‐varying covariates to better understand the dynamic relationships between the variables considered in the study.

In summary, our results show that a peak (i.e., higher levels than usual) in PB is associated with an increase in LS in young adults, and this effect can extend to the next day, but tends not to persist on a weekly basis. Furthermore, although within‐person PB was not associated with next‐day or next‐week HB, it is important to note that some studies using the EMA procedure (i.e., assessments made within a few hours) have found such effects, supporting the idea that this relation may occur when considering shorter time windows of a few hours (e.g., Rinner et al. 2022; Snippe et al. 2018). This suggests that the daily and weekly time span may be too long to capture moment‐to‐moment effects. Overall, this study highlights the importance of promoting daily prosocial behaviors in young adults, suggesting that engaging in such behaviors may be beneficial in a life stage characterized by continuous challenges.

## Author Contributions

**Fulvio Gregori:** conceptualization, data curation, formal analysis, methodology, software, validation, visualization, writing – original draft preparation, writing – review and editing. **Belén Lopéz‐Pérez:** conceptualization, data curation, investigation, methodology, project administration, supervision, writing – original draft preparation, writing – review and editing. **Tyler Colasante:** conceptualization, writing – original draft preparation, writing – review and editing. **Giuseppe Corbelli:** formal analysis, software, validation, visualization; writing – original draft preparation, writing – review and editing. **Tina Malti:** writing – original draft preparation, writing – review and editing. **Manuel Marti‐Vilar:** methodology, project administration, writing – review and editing. **Cristina Di Giusto Valle:** methodology, project administration, writing – review and editing. **Tamara Benito‐Ambrona:** methodology, project administration, writing – review and editing. **Stefania Sette:** methodology, project administration, writing – original draft preparation, writing – review and editing. **Federico Mozzetti:** writing – original draft preparation, writing – review and editing. **Lucia Manfredi:** data curation, writing – original draft preparation, writing – review and editing. **Maria Gerbino:** writing – original draft preparation, writing – review and editing. **Concetta Pastorelli:** writing – original draft preparation, writing – review and editing. **Antonio Zuffianò:** conceptualization, investigation, data curation, investigation, methodology, project administration, validation, supervision, writing – original draft preparation, writing – review and editing.

## Ethics Statement

The ethics committee of the author's institution approved the study. Participants were invited to fill out questionnaires after giving their informed consent.

## Conflicts of Interest

The authors declare no conflicts of interest.

## Supporting information


**Data S1:** jopy70010‐sup‐0001‐Supinfo01.docx.

## Data Availability

Data and analysis scripts used for this article can be accessed at the following link: https://osf.io/s3jv5/?view_only=2ab61425382240f1ae5c12ecfba9824f.
