# Supplementary material for: The Relations Among Prosocial Behavior, Life Satisfaction, and Hedonic Balance Among Young Adults
Source: J Pers. 2025 Aug 8;94(3):419–30. doi: 10.1111/jopy.70010 (PMC13163623; doi:10.1111/jopy.70010)
Supplement: Supplementary file 1 — Data S1: jopy70010‐sup‐0001‐Supinfo01.docx. [file JOPY-94-419-s001.docx]

**Supplementary Material**

**Table S1a**

*Correlations between Prosocial Behavior and Life Satisfaction at each time in the daily sample*

|  | PB_T1 | PB_T2 | PB_T3 | PB_T4 | PB_T5 | PB_T6 | PB_T7 | PB_T8 | PB_T9 | PB_T10 | LS_T1 | LS_T2 | LS_T3 | LS_T4 | LS_T5 | LS_T6 | LS_T7 | LS_T8 | LS_T9 | LS_T10 |
| --- | --- | --- | --- | --- | --- | --- | --- | --- | --- | --- | --- | --- | --- | --- | --- | --- | --- | --- | --- | --- |
| PB_T1 | ─ |  |  |  |  |  |  |  |  |  |  |  |  |  |  |  |  |  |  |  |
| PB_T2 | .585^**^ | ─ |  |  |  |  |  |  |  |  |  |  |  |  |  |  |  |  |  |  |
| PB_T3 | .548^**^ | .708^**^ | ─ |  |  |  |  |  |  |  |  |  |  |  |  |  |  |  |  |  |
| PB_T4 | .488^**^ | .639^**^ | .733^**^ | ─ |  |  |  |  |  |  |  |  |  |  |  |  |  |  |  |  |
| PB_T5 | .486^**^ | .559^**^ | .625^**^ | .739^**^ | ─ |  |  |  |  |  |  |  |  |  |  |  |  |  |  |  |
| PB_T6 | .497^**^ | .595^**^ | .636^**^ | .712^**^ | .724^**^ | ─ |  |  |  |  |  |  |  |  |  |  |  |  |  |  |
| PB_T7 | .445^**^ | .575^**^ | .659^**^ | .657^**^ | .674^**^ | .747^**^ | ─ |  |  |  |  |  |  |  |  |  |  |  |  |  |
| PB_T8 | .430^**^ | .563^**^ | .625^**^ | .668^**^ | .631^**^ | .734^**^ | .751^**^ | ─ |  |  |  |  |  |  |  |  |  |  |  |  |
| PB_T9 | .397^**^ | .531^**^ | .579^**^ | .657^**^ | .636^**^ | .706^**^ | .681^**^ | .735^**^ | ─ |  |  |  |  |  |  |  |  |  |  |  |
| PB_T10 | .408^**^ | .555^**^ | .568^**^ | .677^**^ | .717^**^ | .702^**^ | .717^**^ | .711^**^ | .714^**^ | ─ |  |  |  |  |  |  |  |  |  |  |
| LS_T1 | .189^**^ | .072 | .169^*^ | .095 | .112 | .180^*^ | .108 | .090 | .078 | .096 | ─ |  |  |  |  |  |  |  |  |  |
| LS_T2 | .046 | .204^**^ | .164^*^ | .119 | .143^*^ | .120 | .070 | .045 | -.011 | .172^*^ | .502^**^ | ─ |  |  |  |  |  |  |  |  |
| LS_T3 | .145^*^ | .209^**^ | .303^**^ | .120 | .121 | .160^*^ | .105 | .135^*^ | .059 | .067 | .497^**^ | .547^**^ | ─ |  |  |  |  |  |  |  |
| LS_T4 | .190^**^ | .174^**^ | .244^**^ | .267^**^ | .174^*^ | .207^**^ | .171^*^ | .209^**^ | .131 | .188^**^ | .554^**^ | .511^**^ | .652^**^ | ─ |  |  |  |  |  |  |
| LS_T5 | .172^*^ | .150^*^ | .189^**^ | .207^**^ | .324^**^ | .245^**^ | .169^*^ | .184^**^ | .198^**^ | .181^*^ | .468^**^ | .463^**^ | .489^**^ | .580^**^ | ─ |  |  |  |  |  |
| LS_T6 | .158^*^ | .207^**^ | .178^**^ | .142^*^ | .139^*^ | .322^**^ | .182^**^ | .207^**^ | .103 | .197^**^ | .400^**^ | .440^**^ | .524^**^ | .575^**^ | .590^**^ | ─ |  |  |  |  |
| LS_T7 | .180^**^ | .175^**^ | .201^**^ | .249^**^ | .262^**^ | .314^**^ | .356^**^ | .243^**^ | .207^**^ | .241^**^ | .483^**^ | .411^**^ | .450^**^ | .506^**^ | .540^**^ | .600^**^ | ─ |  |  |  |
| LS_T8 | .173^*^ | .211^**^ | .248^**^ | .266^**^ | .180^**^ | .333^**^ | .272^**^ | .339^**^ | .253^**^ | .202^**^ | .486^**^ | .442^**^ | .564^**^ | .572^**^ | .509^**^ | .666^**^ | .680^**^ | ─ |  |  |
| LS_T9 | .157^*^ | .119 | .143^*^ | .172^*^ | .245^**^ | .272^**^ | .183^**^ | .231^**^ | .273^**^ | .197^**^ | .401^**^ | .436^**^ | .515^**^ | .540^**^ | .534^**^ | .586^**^ | .604^**^ | .678^**^ | ─ |  |
| LS_T10 | .203^**^ | .196^**^ | .248^**^ | .342^**^ | .348^**^ | .370^**^ | .334^**^ | .326^**^ | .291^**^ | .463^**^ | .473^**^ | .501^**^ | .484^**^ | .606^**^ | .518^**^ | .606^**^ | .632^**^ | .647^**^ | .607^**^ | ─ |
| **. Correlation is significant at the 0.01 level (2-tailed). | | | | | | | | | | | | | | | | | | | | |
| *. Correlation is significant at the 0.05 level (2-tailed). | | | | | | | | | | | | | | | | | | | | |

**Table S1b**

*Correlations between Prosocial Behavior and Hedonic Balance at each time in the daily sample*

|  | PB_T1 | PB_T2 | PB_T3 | PB_T4 | PB_T5 | PB_T7 | PB_T8 | PB_T9 | PB_T10 | HB_T1 | HB_T2 | HB_T3 | HB_T4 | HB_T5 | HB_T6 | HB_T7 | HB_T8 | HB_T9 | HB_T10 |
| --- | --- | --- | --- | --- | --- | --- | --- | --- | --- | --- | --- | --- | --- | --- | --- | --- | --- | --- | --- |
| PB_T1 | ─ |  |  |  |  |  |  |  |  |  |  |  |  |  |  |  |  |  |  |
| PB_T2 | .585^**^ | ─ |  |  |  |  |  |  |  |  |  |  |  |  |  |  |  |  |  |
| PB_T3 | .548^**^ | .708^**^ | ─ |  |  |  |  |  |  |  |  |  |  |  |  |  |  |  |  |
| PB_T4 | .488^**^ | .639^**^ | .733^**^ | ─ |  |  |  |  |  |  |  |  |  |  |  |  |  |  |  |
| PB_T5 | .486^**^ | .559^**^ | .625^**^ | .739^**^ | ─ |  |  |  |  |  |  |  |  |  |  |  |  |  |  |
| PB_T7 | .445^**^ | .575^**^ | .659^**^ | .657^**^ | .674^**^ | ─ |  |  |  |  |  |  |  |  |  |  |  |  |  |
| PB_T8 | .430^**^ | .563^**^ | .625^**^ | .668^**^ | .631^**^ | .751^**^ | ─ |  |  |  |  |  |  |  |  |  |  |  |  |
| PB_T9 | .397^**^ | .531^**^ | .579^**^ | .657^**^ | .636^**^ | .681^**^ | .735^**^ | ─ |  |  |  |  |  |  |  |  |  |  |  |
| PB_T10 | .408^**^ | .555^**^ | .568^**^ | .677^**^ | .717^**^ | .717^**^ | .711^**^ | .714^**^ | ─ |  |  |  |  |  |  |  |  |  |  |
| HB_T1 | .087 | .038 | .089 | .054 | .001 | .055 | .033 | .068 | .057 | ─ |  |  |  |  |  |  |  |  |  |
| HB_T2 | .042 | .122^*^ | .095 | .070 | .095 | .070 | -.026 | -.032 | .070 | .603^**^ | ─ |  |  |  |  |  |  |  |  |
| HB_T3 | .090 | .153^*^ | .209^**^ | .086 | .107 | .072 | .032 | .058 | .061 | .477^**^ | .579^**^ | ─ |  |  |  |  |  |  |  |
| HB_T4 | .125 | .153^*^ | .165^*^ | .210^**^ | .156^*^ | .088 | .134^*^ | .150^*^ | .114 | .453^**^ | .509^**^ | .672^**^ | ─ |  |  |  |  |  |  |
| HB_T5 | .064 | .058 | .100 | .051 | .182^**^ | .105 | .102 | .128 | .083 | .312^**^ | .363^**^ | .493^**^ | .564^**^ | ─ |  |  |  |  |  |
| HB_T6 | .084 | .171^*^ | .098 | .122 | .082 | .132 | .147^*^ | .089 | .126 | .476^**^ | .505^**^ | .540^**^ | .586^**^ | .638^**^ | ─ |  |  |  |  |
| HB_T7 | .149^*^ | .134^*^ | .122 | .138^*^ | .159^*^ | .239^**^ | .155^*^ | .125 | .133 | .396^**^ | .461^**^ | .501^**^ | .498^**^ | .537^**^ | .696^**^ | ─ |  |  |  |
| HB_T8 | .074 | .127 | .125 | .130 | .101 | .125 | .184^**^ | .146^*^ | .089 | .438^**^ | .448^**^ | .591^**^ | .585^**^ | .516^**^ | .621^**^ | .611^**^ | ─ |  |  |
| HB_T9 | .073 | .073 | .080 | .062 | .067 | .102 | .048 | .197^**^ | .056 | .391^**^ | .410^**^ | .582^**^ | .569^**^ | .439^**^ | .532^**^ | .520^**^ | .684^**^ | ─ |  |
| HB_T10 | .053 | .047 | .017 | .124 | .125 | .119 | .122 | .159^*^ | .277^**^ | .369^**^ | .442^**^ | .500^**^ | .516^**^ | .424^**^ | .546^**^ | .575^**^ | .639^**^ | .589^**^ | ─ |
| **. Correlation is significant at the 0.01 level (2-tailed). | | | | | | | | | | | | | | | | | | | |
| *. Correlation is significant at the 0.05 level (2-tailed). | | | | | | | | | | | | | | | | | | | |

**Table S2a**

*Correlations between Prosocial Behavior and Life Satisfaction at each time in the weekly sample*

|  | PB_T1 | PB_T2 | PB_T3 | PB_T4 | PB_T5 | LS_T1 | LS_T2 | LS_T3 | LS_T4 | LS_T5 |
| --- | --- | --- | --- | --- | --- | --- | --- | --- | --- | --- |
| PB_T1 | ─ |  |  |  |  |  |  |  |  |  |
| PB_T2 | .585^**^ | ─ |  |  |  |  |  |  |  |  |
| PB_T3 | .560^**^ | .664^**^ | ─ |  |  |  |  |  |  |  |
| PB_T4 | .598^**^ | .584^**^ | .650^**^ | ─ |  |  |  |  |  |  |
| PB_T5 | .505^**^ | .584^**^ | .682^**^ | .812^**^ | ─ |  |  |  |  |  |
| LS_T1 | .079 | .043 | .058 | .175^*^ | .185^*^ | ─ |  |  |  |  |
| LS_T2 | .020 | .258^**^ | .118 | .138 | .147 | .474^**^ | ─ |  |  |  |
| LS_T3 | .074 | .195^*^ | .341^**^ | .318^**^ | .313^**^ | .379^**^ | .517^**^ | ─ |  |  |
| LS_T4 | .078 | .253^**^ | .307^**^ | .441^**^ | .356^**^ | .370^**^ | .428^**^ | .681^**^ | ─ |  |
| LS_T5 | .151 | .265^**^ | .288^**^ | .410^**^ | .451^**^ | .355^**^ | .383^**^ | .599^**^ | .750^**^ | ─ |
| **. Correlation is significant at the 0.01 level (2-tailed). | | | | | | | | | | |
| *. Correlation is significant at the 0.05 level (2-tailed). | | | | | | | | | | |

**Table S2b**

*Correlations between Prosocial Behavior and Hedonic Balance at each time in the weekly sample*

|  | PB_T1 | PB_T2 | PB_T3 | PB_T4 | PB_T5 | HB_T1 | HB_T2 | HB_T3 | HB_T4 | HB_T5 |
| --- | --- | --- | --- | --- | --- | --- | --- | --- | --- | --- |
| PB_T1 | ─ |  |  |  |  |  |  |  |  |  |
| PB_T2 | .585^**^ | ─ |  |  |  |  |  |  |  |  |
| PB_T3 | .560^**^ | .664^**^ | ─ |  |  |  |  |  |  |  |
| PB_T4 | .598^**^ | .584^**^ | .650^**^ | ─ |  |  |  |  |  |  |
| PB_T5 | .505^**^ | .584^**^ | .682^**^ | .812^**^ | ─ |  |  |  |  |  |
| HB_T1 | .209^**^ | .224^**^ | .356^**^ | .321^**^ | .375^**^ | ─ |  |  |  |  |
| HB_T2 | .140 | .196^**^ | .236^**^ | .277^**^ | .291^**^ | .553^**^ | ─ |  |  |  |
| HB_T3 | .154 | .169^*^ | .379^**^ | .293^**^ | .272^**^ | .561^**^ | .539^**^ | ─ |  |  |
| HB_T4 | .151 | .223^**^ | .316^**^ | .400^**^ | .367^**^ | .562^**^ | .594^**^ | .659^**^ | ─ |  |
| HB_T5 | .170^*^ | .171^*^ | .280^**^ | .381^**^ | .439^**^ | .560^**^ | .540^**^ | .544^**^ | .666^**^ | ─ |
| **. Correlation is significant at the 0.01 level (2-tailed). | | | | | | | | | | |
| *. Correlation is significant at the 0.05 level (2-tailed). | | | | | | | | | | |

**Figure S1a**

*Main Results of Random Intercept-Cross Lagged Panel Model (RI-CLPM) Considering Prosocial Behavior and Life Satisfaction in the daily sample*


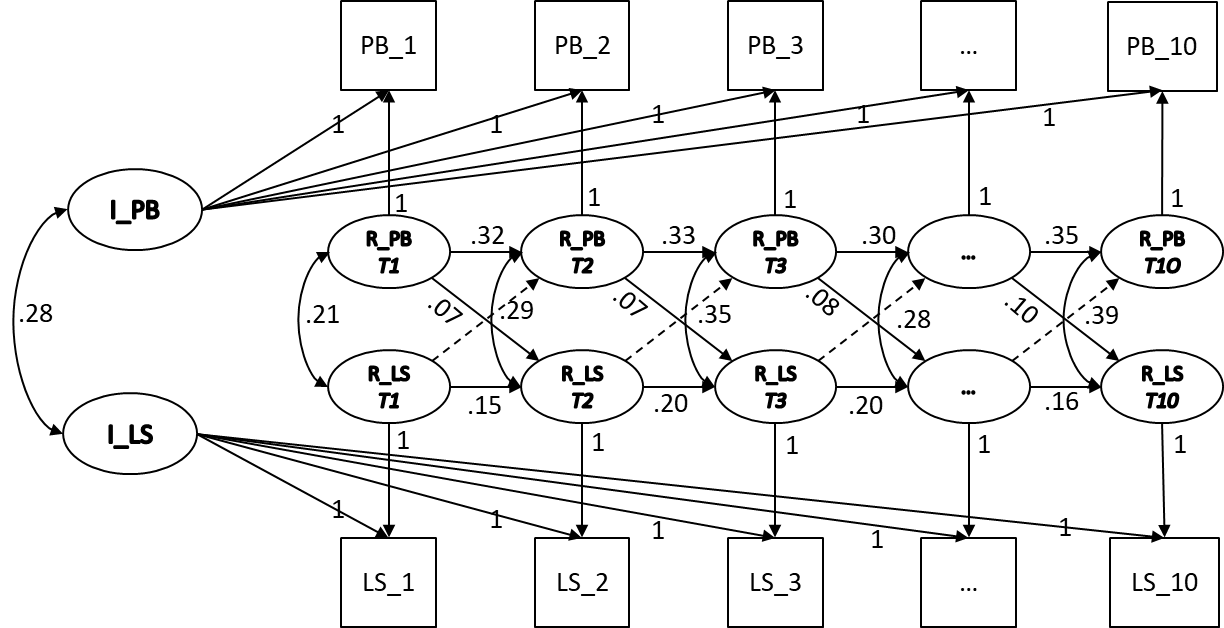
**Figure S1b**

*Main Results of Random Intercept-Cross Lagged Panel Model (RI-CLPM) Considering Prosocial Behavior and Hedonic Balance in the daily sample*


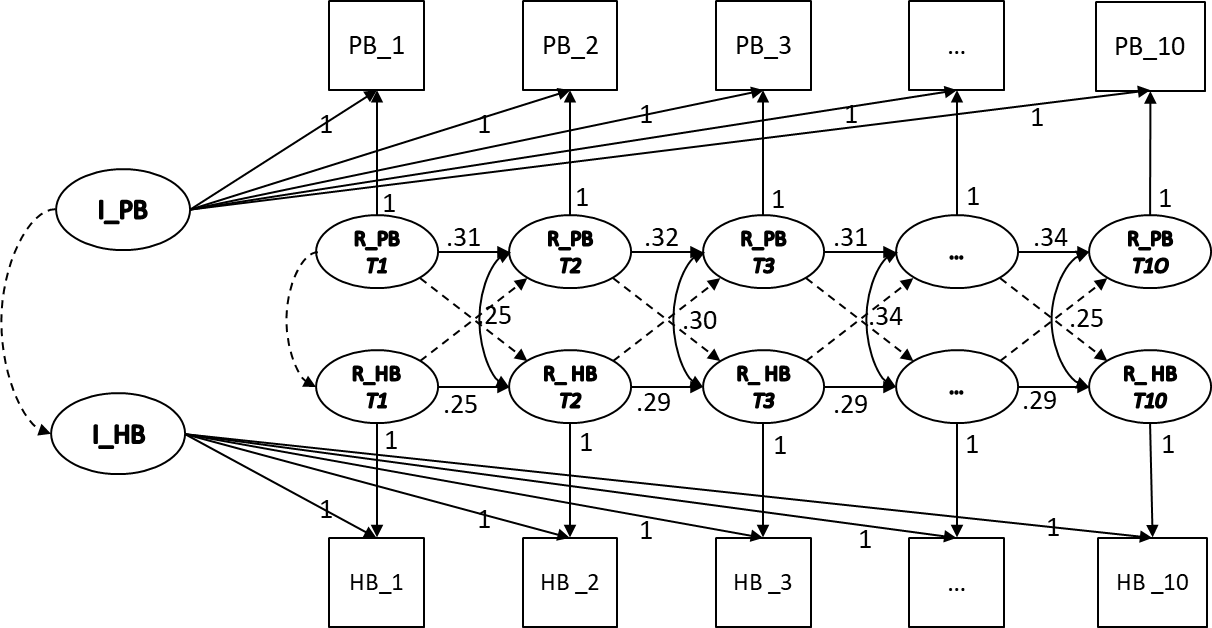


**Figure S2a**

*Main Results of Random Intercept-Cross Lagged Panel Model (RI-CLPM) Considering Prosocial Behavior and Life Satisfaction in the weekly sample*


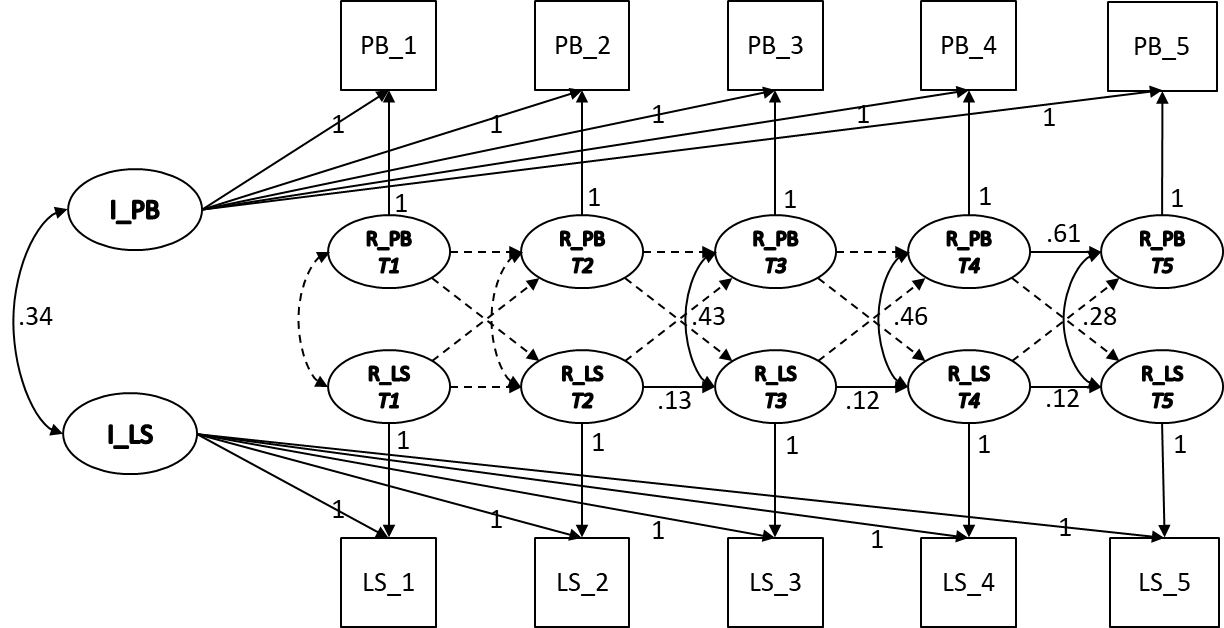


**Figure S2b**

*Main Results of Random Intercept-Cross Lagged Panel Model (RI-CLPM) Considering Prosocial Behavior and Hedonic Balancein the weekly sample*


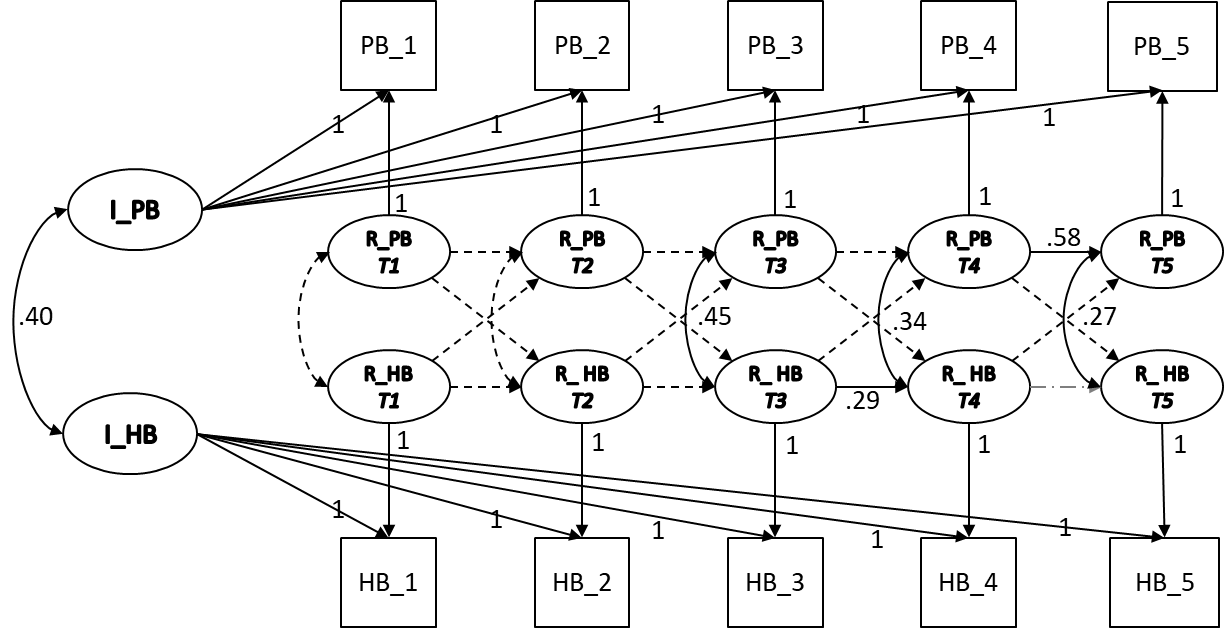


**Table S3**

*Random Intercept Cross Lagged Panel Model (RI_CLPM) in the daily* *sample. The results of the constrained model show the between-person effect and the within-person effects (correlations. carry-over and spillover effects).*

| Parameters | Std(Unst) | SE | p-value | Parameters | Std(Unst) | SE | p-value |
| --- | --- | --- | --- | --- | --- | --- | --- |
| Model 1 |  |  |  | Model 2 |  |  |  |
| Between-level |  |  |  | Between-level |  |  |  |
| I_PB↔I_LS | .280 (.205) | .069 | <.001 | I_PB↔I_HB | .118 (.078) | .071 | .097 |
| Within-level |  |  |  | Within-level |  |  |  |
| *Correlations* |  |  |  | *Correlations* |  |  |  |
| R_PB T1↔R_LS T1 | .207 (.129) | .087 | <.05 | R_PB T1↔R_HB T1 | .068 (.043) | .080 | .394 |
| R_PB T2↔R_LS T2 | .285 (.188) | .034 | <.001 | R_PB T2↔R_HB T2 | .252 (.153) | .025 | <.001 |
| R_PB T3↔R_LS T3 | .345 (.188) | .043 | <.001 | R_PB T3↔R_HB T3 | .296 (.153) | .031 | <.001 |
| R_PB T4↔R_LS T4 | .373 (.188) | .047 | <.001 | R_PB T4↔R_HB T4 | .338 (.153) | .033 | <.001 |
| R_PB T5↔R_LS T5 | .321 (.188) | .038 | <.001 | R_PB T5↔R_HB T5 | .253 (.153) | .029 | <.001 |
| R_PB T6↔R_LS T6 | .330 (.188) | .045 | <.001 | R_PB T6↔R_HB T6 | .299 (.153) | .032 | <.001 |
| R_PB T7↔R_LS T7 | .304 (.188) | .043 | <.001 | R_PB T7↔R_HB T7 | .255 (.153) | .031 | <.001 |
| R_PB T8↔R_LS T8 | .331 (.188) | .038 | <.001 | R_PB T8↔R_HB T8 | .257 (.153) | .028 | <.001 |
| R_PB T9↔R_LS T9 | .280 (.188) | .036 | <.001 | R_PB T9↔R_HB T9 | .249 (.153) | .032 | <.001 |
| R_PB T10↔R_LS T10 | .386 (.188) | .044 | <.001 | R_PB T10↔R_HB T10 | .309 (.153) | .034 | <.001 |
| *Carry-over* |  |  |  | *Carry-over* |  |  |  |
| R_PB T1→R_PB T2 | .320 (.307) | .047 | <.001 | R_PB T1→R_PB T2 | .311 (.301) | .045 | <.001 |
| R_PB T2→R_PB T3 | .330 (.307) | .055 | <.001 | R_PB T2→R_PB T3 | .321 (.301) | .055 | <.001 |
| R_PB T3→R_PB T4 | .300 (.307) | .057 | <.001 | R_PB T3→R_PB T4 | .314 (.301) | .057 | <.001 |
| R_PB T4→R_PB T5 | .294 (.307) | .051 | <.001 | R_PB T4→R_PB T5 | .277 (.301) | .047 | <.001 |
| R_PB T5→R_PB T6 | .310 (.307) | .056 | <.001 | R_PB T5→R_PB T6 | .298 (.301) | .052 | <.001 |
| R_PB T6→R_PB T7 | .278 (.307) | .049 | <.001 | R_PB T6→R_PB T7 | .272 (.301) | .049 | <.001 |
| R_PB T7→R_PB T8 | .312 (.307) | .052 | <.001 | R_PB T7→R_PB T8 | .308 (.301) | .049 | <.001 |
| R_PB T8→R_PB T9 | .288 (.307) | .056 | <.001 | R_PB T8→R_PB T9 | .289 (.301) | .055 | <.001 |
| R_PB T9→R_PB T10 | .352 (.307) | .052 | <.001 | R_PB T9→R_PB T10 | .337 (.301) | .049 | <.001 |
| R_LS T1→R_LS T2 | .150 (.177) | .029 | <.001 | R_HB T1→R_HB T2 | .247 (.264) | .036 | <.001 |
| R_LS T2→R_LS T3 | .197 (.177) | .041 | <.001 | R_HB T2→R_HB T3 | .285 (.264) | .040 | <.001 |
| R_LS T3→R_LS T4 | .198 (.177) | .041 | <.001 | R_HB T3→R_HB T4 | .289 (.264) | .041 | <.001 |
| R_LS T4→R_LS T5 | .161 (.177) | .032 | <.001 | R_HB T4→R_HB T5 | .221 (.264) | .033 | <.001 |
| R_LS T5→R_LS T6 | .179 (.177) | .038 | <.001 | R_HB T5→R_HB T6 | .304 (.264) | .043 | <.001 |
| R_LS T6→R_LS T7 | .182 (.177) | .038 | <.001 | R_HB T6→R_HB T7 | .255 (.264) | .040 | <.001 |
| R_LS T7→R_LS T8 | .187 (.177) | .041 | <.001 | R_HB T7→R_HB T8 | .256 (.264) | .034 | <.001 |
| R_LS T8→R_LS T9 | .162 (.177) | .033 | <.001 | R_HB T8→R_HB T9 | .266 (.264) | .043 | <.001 |
| R_LS T9→R_LS T10 | .206 (.177) | .042 | <.001 | R_HB T9→R_HB T10 | .285 (.264) | .036 | <.001 |
| *Spillover* |  |  |  | *Spillover* |  |  |  |
| R_PB T1→R_LS T2 | .068 (.132) | .028 | <.05 | R_PB T1→R_HB T2 | .006 (.011) | .026 | .804 |
| R_PB T2→R_LS T3 | .072 (.132) | .032 | <.05 | R_PB T2→R_HB T3 | .007 (.011) | .027 | .805 |
| R_PB T3→R_LS T4 | .075 (.132) | .033 | <.05 | R_PB T3→R_HB T4 | .007 (.011) | .028 | .805 |
| R_PB T4→R_LS T5 | .070 (.132) | .030 | <.05 | R_PB T4→R_HB T5 | .006 (.011) | .022 | .805 |
| R_PB T5→R_LS T6 | .073 (.132) | .031 | <.05 | R_PB T5→R_HB T6 | .007 (.011) | .028 | .805 |
| R_PB T6→R_LS T7 | .074 (.132) | .033 | <.05 | R_PB T6→R_HB T7 | .007 (.011) | .027 | .805 |
| R_PB T7→R_LS T8 | .086 (.132) | .038 | <.05 | R_PB T7→R_HB T8 | .007 (.011) | .030 | .805 |
| R_PB T8→R_LS T9 | .077 (.132) | .034 | <.05 | R_PB T8→R_HB T9 | .007 (.011) | .029 | .805 |
| R_PB T9→R_LS T10 | .096 (.132) | .042 | <.05 | R_PB T9→R_HB T10 | .008 (.011) | .033 | .805 |
| R_LS T1→R_PB T2 | -.008 (-.005) | .034 | .804 | R_HB T1→R_PB T2 | .005 (.003) | .027 | .854 |
| R_LS T2→R_PB T3 | -.011 (-.005) | .043 | .802 | R_HB T2→R_PB T3 | .006 (.003) | .031 | .855 |
| R_LS T3→R_PB T4 | -.009 (-.005) | .038 | .804 | R_HB T3→R_PB T4 | .005 (.003) | .030 | .854 |
| R_LS T4→R_PB T5 | -.008 (-.005) | .033 | .804 | R_HB T4→R_PB T5 | .005 (.003) | .025 | .854 |
| R_LS T5→R_PB T6 | -.009 (-.005) | .036 | .804 | R_HB T5→R_PB T6 | .005 (.003) | .029 | .854 |
| R_LS T6→R_PB T7 | -.008 (-.005) | .033 | .803 | R_HB T6→R_PB T7 | .004 (.003) | .023 | .854 |
| R_LS T7→R_PB T8 | -.008 (-.005) | .032 | .804 | R_HB T7→R_PB T8 | .004 (.003) | .024 | .854 |
| R_LS T8→R_PB T9 | -.007 (-.005) | .029 | .804 | R_HB T8→R_PB T9 | .004 (.003) | .024 | .854 |
| R_LS T9→R_PB T10 | -.009 (-.005) | .036 | .804 | R_HB T9→R_PB T10 | .005 (.003) | .027 | .854 |

*Note*. I_LS=Intercept of Life Satisfaction; I_PB=Intercept of Prosocial Behavior; I_HB=Intercept of Hedonic Balance; R_PB_T1…R_PB_T10= Within component of Prosocial Behavior from day 1. to day10; R_LS_T1… R_LS_T10=Within component of Life Satisfaction from day 1 to day 1; R_HB_T1…R_HB_T10= Within component of Hedonic Balance from day 1. to day10.

**Table S4**

*Random Intercept Cross Lagged Panel Model (RI_CLPM) in the weekly sample. The results of the unconstrained model show the between-person effect and the within-person effects (correlations. carry-over and spillover effects).*

| Parameters | Std(Unst) | SE | p-value | Parameters | Std(Unst) | SE | p-value |
| --- | --- | --- | --- | --- | --- | --- | --- |
| Model 3 |  |  |  | Model 4 |  |  |  |
| Between level |  |  |  | Between level |  |  |  |
| I_PB↔I_LS | .335 (.145) | .129 | <.01 | I_PB↔I_HB | .397 (.201) | .095 | <.001 |
| Within level |  |  |  | Within level |  |  |  |
| *Correlations* |  |  |  | *Correlations* |  |  |  |
| R_PB T1↔R_LS T1 | -.211 (-.083) | .180 | .241 | R_PB T1↔R_HB T1 | -.271 (-.086) | .209 | .196 |
| R_PB T2↔R_LS T2 | .199 (.075) | .189 | .292 | R_PB T2↔R_HB T2 | -.270 (-.071) | .324 | .405 |
| R_PB T3↔R_LS T3 | .430 (.228) | .082 | <.001 | R_PB T3↔R_HB T3 | .454 (.215) | .091 | <.001 |
| R_PB T4↔R_LS T4 | .459 (.240) | .088 | <.001 | R_PB T4↔R_HB T4 | .343 (.163) | .100 | <.001 |
| R_PB T5↔R_LS T5 | .282 (.125) | .067 | <.001 | R_PB T5↔R_HB T5 | .272 (.119) | .096 | <.01 |
| *Carry-over* |  |  |  | *Carry-over* |  |  |  |
| R_PB T1→R_PB T2 | -.333 (-.362) | .264 | .206 | R_PB T1→R_PB T2 | -.353 (-.350) | .322 | .273 |
| R_PB T2→R_PB T3 | .214 (.286) | .138 | .120 | R_PB T2→R_PB T3 | .114 (.152) | .173 | .511 |
| R_PB T3→R_PB T4 | .187 (.197) | .141 | .184 | R_PB T3→R_PB T4 | .195 (.213) | .159 | .222 |
| R_PB T4→R_PB T5 | .608 (.718) | .097 | <.001 | R_PB T4→R_PB T5 | .581 (.679) | .088 | <.001 |
| R_LS T1→R_LS T2 | .125 (.125) | .176 | .476 | R_HB T1→R_HB T2 | -.102 (-.097) | .220 | .641 |
| R_LS T2→R_LS T3 | .324 (.329) | .129 | <.05 | R_HB T2→R_HB T3 | .108 (.130) | .127 | .394 |
| R_LS T3→R_LS T4 | .522 (.549) | .116 | <.01 | R_HB T3→R_HB T4 | .288 (.282) | .131 | <.05 |
| R_LS T4→R_LS T5 | .553 (.549) | .116 | <.001 | R_HB T4→R_HB T5 | .219 (.224) | .137 | .110 |
| *Spillover* |  |  |  | *Spillover* |  |  |  |
| R_PB T1→R_LS T2 | -.234 (-.744) | .173 | .177 | R_PB T1→R_HB T2 | -.286 (-.646) | .227 | .208 |
| R_PB T2→R_LS T3 | .096 (.288) | .110 | .379 | R_PB T2→R_HB T3 | .010 (.027) | .133 | .940 |
| R_PB T3→R_LS T4 | .017 (.040) | .107 | .874 | R_PB T3→R_HB T4 | .055 (.111) | .141 | .694 |
| R_PB T4→R_LS T5 | .146 (.324) | .088 | .099 | R_PB T4→R_HB T5 | .253 (.477) | .128 | <.05 |
| R_LS T1→R_PB T2 | -.283 (-.096) | .208 | .175 | R_HB T1→R_PB T2 | -.275 (-.115) | .280 | .326 |
| R_LS T2→R_PB T3 | .037 (.017) | .128 | .771 | R_HB T2→R_PB T3 | .067 (.039) | .173 | .701 |
| R_LS T3→R_PB T4 | .165 (.078) | .120 | .170 | R_HB T3→R_PB T4 | .032 (.017) | .153 | .836 |
| R_LS T4→R_PB T5 | .070 (.037) | .095 | .462 | R_HB T4→R_PB T5 | .115 (.073) | .092 | .213 |

*Note*. I_LS=Intercept of Life Satisfaction; I_PB=Intercept of Prosocial Behavior; I_HB=Intercept of Hedonic Balance; R_PB_T1…R_PB_T5= Within component of Prosocial Behavior from week 1. to week 5; R_LS_T1… R_LS_T5=Within component of Life Satisfaction from week 1 to week 5; R_HB_T1… R_HB_T5=Within component of Hedonic Balance from week 1 to week 5.
